# The Role of Fear of Negative Evaluation and Loneliness in Linking Insecure Attachment to Social Media Addiction: Evidence from Chinese University Students

**DOI:** 10.3390/brainsci15080843

**Published:** 2025-08-07

**Authors:** Di Xu, Ruoxi He

**Affiliations:** 1 School of Journalism and Information Communication, Huazhong University of Science and Technology, Wuhan 430000, China; heruoxi@hust.edu.cn; 2 Philosophy and Social Sciences Laboratory of Big Data and National Communication Strategy, Ministry of Education, Wuhan 430000, China

**Keywords:** social media addiction, problematic social media use, attachment anxiety, attachment avoidance, fear of negative evaluation, social anxiety, loneliness, serial mediation

## Abstract

Background and Objectives: With the widespread integration of digital media into daily life, social media addiction (SMA) has become a growing concern for university students’ mental health. Based on attachment theory, this study examined how attachment anxiety and avoidance influence SMA through fear of negative evaluation (FNE) and loneliness. Methods: A sample of 400 Chinese university students completed the 16-item short version of the Experiences in Close Relationships Scale (ECR), the 8-item Brief Fear of Negative Evaluation Scale (BFNE), the 6-item Revised UCLA Loneliness Scale–Short Form (RULS-6), and the 6-item Bergen Social Media Addiction Scale (BSMAS). Using the PROCESS macro (Model 6), a chained mediation model was tested. Results: Attachment anxiety positively predicts SMA (*β* = 0.42); the chained mediation pathway through FNE and loneliness accounts for ab = 0.06 of this effect, alongside additional single-mediator paths. In contrast, attachment avoidance shows a weaker total effect (*β* = −0.08) and a small negative chained mediation effect (*ab* = −0.02), offset by opposing single-mediator paths via FNE (negative) and loneliness (positive), resulting in a nonsignificant total indirect effect. Discussion: These findings suggest that in the Chinese cultural context, where social evaluation and belonging are emphasized, insecure attachment may heighten emotional reliance on social media. This study elucidates the socio-emotional mechanisms underlying SMA and extends the application of attachment theory to the digital media environment.

## 1. Introduction

With the deep integration of digital technologies into everyday life, social media has transformed from a tool for information exchange into a psychological space for identity construction, social connection, and emotional regulation, especially among university students, who are often considered “digital natives” [1,2]. In China, platforms such as WeChat, Weibo, and Douyin have created a highly mediatized environment that reshapes individuals’ social rhythms, cognitive frameworks, and emotional expression [3].

Yet, this pervasive digital immersion is not always adaptive. For individuals with vulnerable psychological structures, social media use may escalate into social media addiction (SMA), a behavioral addiction marked by compulsive engagement, impaired self-control, and significant real-life dysfunction. Core symptoms include salience, mood modification, tolerance, withdrawal, conflict, and relapse [4]. Increasingly, SMA has been recognized as a global mental health concern linked to anxiety, depression, attention deficits, and academic decline [5,6,7].

While prior studies have examined SMA through the lenses of personality traits (e.g., self-esteem and neuroticism), usage motivations (e.g., identity seeking and escapism), and media habits [8,9,10,11,12], fewer have addressed the deeper psychological structures and emotional dynamics underlying SMA, particularly within the Chinese socio-cultural context, where evaluative pressure and interpersonal sensitivity are pronounced. In such environments, relational patterns and emotional regulation may be central to understanding compulsive media use.

This study adopts attachment theory as its conceptual framework. According to this theory, early caregiving relationships shape enduring patterns of emotion regulation and interpersonal expectations [13,14]. Insecure attachment in adulthood typically manifests as attachment anxiety (hypersensitivity to relational uncertainty) or attachment avoidance (discomfort with intimacy and emotional suppression). These patterns have been linked to psychological maladjustment [15] and may predispose individuals to seek emotional compensation or substitute connection through social media [9,16].

Within this framework, fear of negative evaluation (FNE) and loneliness are proposed as key mediators. FNE reflects heightened sensitivity to others’ judgments, often co-occurring with social anxiety [17], while loneliness arises from unmet relational needs and emotional disconnection [18]. Both factors may diminish real-world social motivation and intensify reliance on digital platforms for affirmation and relief [19,20,21]. Accordingly, this study proposes a chained mediation model in which insecure attachment predicts SMA through FNE and loneliness. By testing this model among Chinese university students, we aim to illuminate the psychological pathways of SMA and inform culturally sensitive intervention strategies.

## 2. Literature Review and Research Hypotheses

### 2.1. Insecure Attachment and Social Media Addiction

Adult attachment styles are generally categorized as secure or insecure, with the latter comprising two primary subtypes: attachment anxiety and attachment avoidance [22]. Individuals high in attachment anxiety tend to exhibit excessive dependency and hypersensitivity to rejection, driven by persistent doubts regarding the availability of attachment figures [23]. Those with attachment avoidance suppress attachment needs, avoid emotional disclosure, and emphasize autonomy, reflecting resistance to emotional dependency [24,25]. Despite their behavioral differences, both styles represent impairments in relational regulation and emotional coping.

Neuroscientific evidence supports these distinctions. Anxiously attached individuals show amygdala hyperactivation in response to emotional threats and reduced prefrontal regulation, resulting in heightened emotional reactivity and diminished cognitive control [26]. Conversely, avoidantly attached individuals exhibit blunted neural responses to emotional stimuli, reflecting active suppression of emotional processing [27]. Thus, attachment styles are grounded in neuroregulatory mechanisms.

Attachment theory offers a foundational framework for understanding the formation of close relationships and emotional regulation mechanisms. It posits that early relational experiences with caregivers are internalized through internal working models, forming stable psychological structures that shape one’s self-perception, relational expectations, and emotional response patterns [13,14]. These models serve as the cognitive basis for managing uncertainty and social pressure, particularly in intimate relationships.

With the emotionalization of social media, individuals’ digital interaction patterns increasingly reflect their underlying attachment dynamics [28]. Social media has evolved into a psychological space for identity affirmation, emotional regulation, and relational compensation [1]. For those with insecure attachment, it may serve as a substitute attachment system, offering perceived connection and emotional relief [29].

From this perspective, social media addiction (SMA) can be conceptualized as a structural attachment substitution, wherein unmet relational needs are partially fulfilled through digital interaction [30,31]. Anxiously attached individuals may be drawn to the immediacy and responsiveness of social media, which satisfies their need for reassurance and validation [9,32]. Over time, this reliance may evolve into structural dependence, as social media becomes a primary source of emotional regulation and self-worth [33,34].

Avoidantly attached individuals, in contrast, tend to exhibit a dual pattern. Avoidantly attached individuals, by contrast, tend to exhibit a dual pattern. On the one hand, their discomfort with emotional closeness may reduce their motivation for social media use and lower the risk of addiction [28]. On the other hand, the non-face-to-face, controllable, and emotionally detached nature of digital platforms offers a low-risk environment for maintaining minimal social contact [11]. Their engagement is often functional and strategic, driven by a desire for autonomy and control rather than emotional connection [35,36].

This suggests that avoidant users tend to adopt a “low emotional engagement–high control” strategy, marked by selective and emotionally distant media use. In comparison, anxious users are more likely to develop compensatory dependence, seeking external affirmation to manage relational insecurity. These distinct pathways underscore how different attachment dimensions can each, albeit through divergent mechanisms, contribute to heightened social media addiction.

Within the Chinese cultural context, where social evaluation and group belonging are emphasized and parental psychological control tends to be stronger, these dynamics may be further intensified [37,38,39]. Anxiously attached individuals may experience heightened pressure to be seen and affirmed, while avoidantly attached individuals may navigate social expectations through controlled digital interaction. Thus, attachment styles significantly shape social media engagement by influencing emotion regulation, social motivation, and perceived relational risk.

In summary, attachment theory provides a robust framework for understanding the psychological mechanisms underlying SMA. Accordingly, the following hypotheses are proposed:

**H1a:**  *Attachment anxiety is positively associated with social media addiction.*

**H1b:**  *Attachment avoidance is positively associated with social media addiction.*

### 2.2. The Mediating Role of Fear of Negative Evaluation

In exploring the pathway through which insecure attachment contributes to social media addiction (SMA), fear of negative evaluation (FNE) may serve as a key proximal social-emotional mediator. FNE is generally defined as a persistent concern about receiving negative judgments from others, a tendency to avoid social situations, and an anticipatory fear of rejection. It is considered a core component of social anxiety [17], and has been consistently linked to depressive symptoms, avoidance behaviors, and impairments in social functioning [40,41].

From a neurobiological perspective, the emergence of FNE is closely tied to dysfunctions in the brain’s social-emotional processing systems. Studies have shown that individuals with high levels of FNE exhibit hyperactivation of the amygdala when facing social threats, making them highly sensitive to potential negative feedback [42]. Additionally, the anterior cingulate cortex (ACC), a region responsible for regulating social conflict and emotional exclusion, is functionally compromised in these individuals, thereby weakening their capacity for cognitive regulation of emotional stress [43]. These findings suggest that FNE is not merely a cognitive manifestation of social anxiety, but also reflects an emotion-processing dysfunction rooted in neural mechanisms.

At the psychological structural level, attachment theory offers a systematic explanation for the development of FNE. Individuals with attachment anxiety tend to be highly sensitive to relational instability and often doubt their own worthiness of love and acceptance. This leads to the internalization of a negative self-schema, such as “I am not good enough” [25]. Their heavy dependence on external validation amplifies emotional responses to evaluative threats, resulting in elevated levels of FNE [44]. Although avoidantly attached individuals typically avoid intimacy and emotional disclosure on a behavioral level, they may still harbor heightened sensitivity to identity threats at a deeper level. This form of defensive evaluative anxiety reflects an underlying sense of insecurity within their self-protective mechanisms [45]. Empirical evidence supports this view, showing that attachment avoidance is positively associated with social anxiety [46,47], with FNE constituting a central indicator of this emotional difficulty [48].

Based on the theoretical and empirical literature, the following hypotheses are proposed:

**H2a:**  *Attachment anxiety is positively associated with fear of negative evaluation.*

**H2b:**  *Attachment avoidance is positively associated with fear of negative evaluation.*

Beyond being an emotional derivative of the attachment system, fear of negative evaluation (FNE) is also recognized as an important predictor of social media addiction (SMA). On the one hand, social media platforms offer anonymous, asynchronous, and controllable communication environments, allowing individuals with high FNE to avoid the uncontrollable negative evaluations often encountered in face-to-face interactions, and to construct more idealized self-presentations [49,50]. Such environments not only reduce perceived social threats but also provide a safe space for low-risk emotional expression, which helps alleviate the psychological discomfort associated with social anxiety [20]. This mechanism aligns closely with both the social compensation hypothesis [51] and the compensatory Internet use theory [52], both of which emphasize the emotion regulation substitute role of digital media.

On the other hand, even in the relatively low-pressure evaluative context of social media, individuals with high FNE remain highly sensitive to online feedback, such as likes, comments, and private messages. They often engage in behaviors such as frequent message checking, repeated editing of posts, and deleting comments, in order to minimize the possibility of negative evaluation [19]. These behaviors, in turn, reinforce their emotional dependence on the platform, trapping them in a self-reinforcing cycle in which anxiety leads to increased usage, which subsequently results in greater dependence.

It is important to note that the impact of FNE on SMA goes beyond psychological avoidance; it reflects a strong emotional compensation motive. High-FNE individuals are not merely evading social interaction, but actively seeking a form of “controlled visibility” in the mediated environment to reduce the anxiety associated with real-world evaluation. This emotion regulation pathway serves as both a defensive mechanism and a potential risk factor for addiction.

Therefore, FNE functions not only as an emotional manifestation of insecure attachment but also as a critical bridging variable linking insecure attachment to SMA. Based on these theoretical and empirical insights, the following hypotheses are proposed:

**H3:**  *Fear of negative evaluation is positively associated with social media addiction.*

**H4a:**  *Fear of negative evaluation mediates the relationship between attachment anxiety and social media addiction.*

**H4b:**  *Fear of negative evaluation mediates the relationship between attachment avoidance and social media addiction.*

### 2.3. The Mediating Role of Loneliness and Its Serial Pathway with FNE

Building on previous research that has identified fear of negative evaluation (FNE) as a key predictor of individuals’ social behavior and media dependency, loneliness, as a deeper and more pervasive social-emotional experience, may also play a crucial role in the mechanism through which insecure attachment contributes to social media addiction (SMA). Loneliness is typically defined as a subjective negative emotional state that arises when an individual’s need for social connection is unmet, and is fundamentally characterized by the perceived absence of reciprocated intimacy [18,53]. From the perspective of attachment theory, loneliness reflects the emotional consequences of attachment regulation failure, representing a dysfunction in the affective adaptation system of close relationships.

Specifically, individuals with attachment anxiety, due to their heightened dependence on relational responsiveness, are prone to experience feelings of rejection and neglect when sustained feedback is unavailable, which in turn triggers significant loneliness [54]. Although avoidantly attached individuals tend to suppress emotional expression and vulnerability at the behavioral level, their repressed attachment needs, when chronically unfulfilled, may also accumulate into emotional emptiness and social disconnection [14]. Therefore, loneliness may represent a shared emotional outcome of both forms of insecure attachment, albeit arising from distinct psychological mechanisms.

**H5a:**  *Attachment anxiety is positively associated with loneliness.*

**H5b:**  *Attachment avoidance is positively associated with loneliness.*

From a neuroscientific perspective, loneliness is closely associated with hypoactivity in the brain’s reward system. Studies have shown that lonely individuals exhibit significantly reduced activation in the nucleus accumbens when exposed to socially positive stimuli (e.g., smiles and praise), indicating diminished sensitivity to social rewards and a reduced capacity for emotional satisfaction through real-life interactions [55]. This reward-deficit state may lead individuals to turn to virtual platforms, such as social media, in search of immediate feedback and compensatory connections [56]. However, digital interactions often lack emotional depth and relational stability, which may result in a vicious cycle of loneliness, compensation, and disappointment [57].

Empirical studies have consistently confirmed the critical role of loneliness in the development of SMA. Individuals experiencing loneliness, due to the absence of real-world social support, are more likely to use social media as a substitute means of emotional comfort, affirmation, and companionship, which in turn increases both usage frequency and psychological dependence [58,59]. Yet, the superficial and unstable nature of virtual interactions may exacerbate feelings of loneliness and intensify media dependency [3,60,61,62].

**H6:**  *Loneliness is positively associated with social media addiction.*

Further, loneliness is not only an emotional outcome of attachment system dysfunction but may also serve as a mediating mechanism in the pathway from attachment style to social media addiction (SMA) [63]. Individuals with attachment anxiety frequently encounter emotional disappointment in real-life relationships, which increases feelings of loneliness and, in turn, leads them to seek compensatory connections through social media. Conversely, avoidantly attached individuals, while suppressing emotional needs over time, may engage in strategic media use to maintain a minimal level of emotional connection.

**H7a:**  *Loneliness mediates the relationship between attachment anxiety and social media addiction.*

**H7b:**  *Loneliness mediates the relationship between attachment avoidance and social media addiction.*

It is important to emphasize that FNE and loneliness are not two independent parallel mediators, but rather exhibit a sequential and causal relationship. Prior research has shown that FNE undermines individuals’ social motivation and willingness to express emotions, thereby limiting their ability to form authentic and intimate relationships, which indirectly contributes to heightened loneliness [64,65]. In other words, FNE not only reflects fear of evaluation but may also diminish social connectedness at the emotional level, forming an emotional progression from FNE to loneliness [21,49,66].

**H8:**  *Fear of negative evaluation is positively associated with loneliness.*

Based on this, FNE and loneliness may together constitute a serial mediation mechanism, wherein each layer of social-emotional distress contributes to the next, ultimately increasing the risk of social media dependence. This pathway not only reveals how insecure attachment influences SMA through multiple emotional processes, but also reflects the psychological adaptation logic of individuals in mediated environments.

**H9a:**  *Fear of negative evaluation and loneliness sequentially mediate the relationship between attachment anxiety and social media addiction.*

**H9b:**  *Fear of negative evaluation and loneliness sequentially mediate the relationship between attachment avoidance and social media addiction.*

Within the context of Chinese culture, this mechanism pathway may be further intensified. Collectivist cultural values, which emphasize group belonging, social evaluation, and the maintenance of “face”, tend to foster heightened self-monitoring in response to external judgments [67]. Among Chinese university students, frequent engagement in social media activities, such as self-presentation, social interaction, and feedback-seeking behaviors, is often driven by fear of negative evaluation (FNE) [68]. At the same time, intense academic pressure, family expectations, and limited access to real-world social resources may exacerbate feelings of loneliness, thereby reinforcing emotionally motivated and compensatory use of media platforms [69]. This suggests that under specific cultural conditions, the serial pathway from FNE to loneliness not only demonstrates cross-cultural generalizability but also carries unique contextual significance within local socio-cultural environments.

In summary, loneliness is not only an emotional manifestation of attachment system dysfunction but also emerges as part of a progressive emotional chain influenced by FNE, ultimately expressed through media use behavior. This mechanism model offers a multi-level, systematic theoretical explanation for understanding how insecure attachment contributes to SMA and provides a psychological and emotional basis for developing future culture-sensitive intervention strategies.

**Figure 1:**
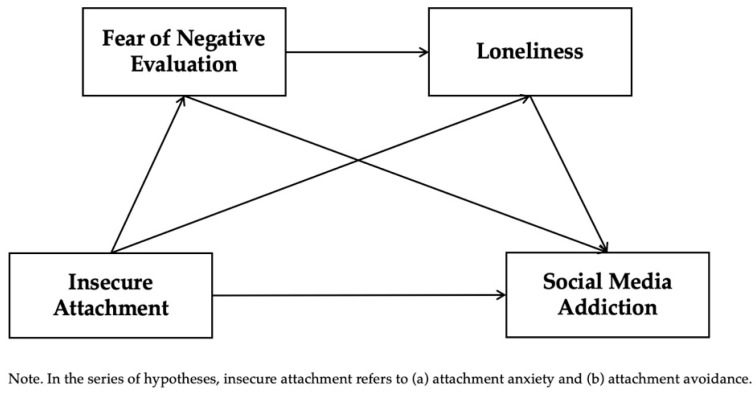
The serial mediation model hypothesized in the present study.

Building on the above, this study proposes a multi-path mediation model grounded in attachment theory, in which insecure attachment influences social media addiction (SMA) through both direct and indirect emotional mechanisms. Specifically, fear of negative evaluation (FNE) functions as a proximal social-cognitive mediator, while loneliness reflects a deeper emotional consequence of attachment system dysfunction. The model incorporates both parallel and sequential mediation pathways, positing that FNE increases susceptibility to loneliness, which in turn reinforces compensatory media use. Together, these constructs form a serial emotional chain from attachment insecurity to SMA. This framework not only elucidates the layered psychological mechanisms linking attachment and media dependency but also highlights the culturally shaped dynamics of emotional vulnerability and digital coping among Chinese university students. The hypothesized model is illustrated in Figure 1. Accordingly, the study proposes the following hypotheses:

**H1:**  *(a) Attachment anxiety and (b) attachment avoidance are positively associated with social media addiction.*

**H2:**  *(a) Attachment anxiety and (b) attachment avoidance are positively associated with fear of negative evaluation.*

**H3:**  *Fear of negative evaluation is positively associated with social media addiction.*

**H4:**  *Fear of negative evaluation mediates the relationship between (a) attachment anxiety, (b) attachment avoidance, and social media addiction.*

**H5:**  *(a) Attachment anxiety and (b) attachment avoidance are positively associated with loneliness.*

**H6:**  *Loneliness is positively associated with social media addiction.*

**H7:**  *Loneliness mediates the relationship between (a) attachment anxiety, (b) attachment avoidance, and social media addiction.*

**H8:**  *Fear of negative evaluation is positively associated with loneliness.*

**H9:**  *Fear of negative evaluation and loneliness sequentially mediate the relationship between (a) attachment anxiety, (b) attachment avoidance, and social media addiction.*

## 3. Materials and Methods

### 3.1. Participants

Among 400 participants, 48.25% were male (*n* = 193) and 51.75% female (*n* = 207), aged 18–27 years (*M* = 22.28, *SD* = 1.93). In total, 58.25% came from urban areas, 31.75% were only children, and 38.00% were in romantic relationships. Educational levels included Junior College (6.00%), Undergraduate (69.00%), Master’s (24.00%), and Doctoral (1.00%). Participants represented 13 academic disciplines, including literature, law, medicine, science, and engineering. Regarding academic stress, 55.25% reported high levels, and 21.00% reported moderate levels. Participants’ mean daily social media usage was 3.32 (*SD* = 0.92), based on a 5-point scale coding of self-reported usage options (1 = “<1 h”, 2 = “1–2 h”, 3 = “2–4 h”, 4 = “4–6 h”, 5 = “≥6 h”), as shown in Table 1.

### 3.2. Instruments

#### 3.2.1. Insecure Attachment

Insecure attachment was assessed using a 16-item short version of the Experiences in Close Relationships Scale (ECR) [23], which measures two dimensions: attachment anxiety (e.g., “I get upset or angry if I don’t receive enough attention and care from my partner”) and attachment avoidance (e.g., “Generally, I don’t like to let my partner know what I’m really feeling inside”). Items were rated on a 5-point Likert scale (1 = strongly disagree, 5 = strongly agree). The Chinese version demonstrated good psychometric properties among Chinese college students [70]. In this study, McDonald’s ω was 0.93 for attachment anxiety and 0.92 for attachment avoidance.

#### 3.2.2. Fear of Negative Evaluation

Fear of negative evaluation (FNE) was measured using the 8-item Brief Fear of Negative Evaluation Scale (BFNE) [71], rated on a 5-point scale (1 = Not at all like me, 5 = Very much like me). A sample item is “I often worry that others will notice my shortcomings”. The Chinese version was validated using college student samples [72]. In this study, McDonald’s ω was 0.95.

#### 3.2.3. Loneliness

Loneliness was assessed with the 6-item Revised UCLA Loneliness Scale–Short Form (RULS-6) [73], using a 5-point scale (1 = Never, 5 = Always). An example item is “Lack of companionship”. The Chinese version showed strong reliability and validity [74]. In the present sample, McDonald’s ω was 0.93.

#### 3.2.4. Social Media Addiction

Social media addiction (SMA) was measured using the 6-item Bergen Social Media Addiction Scale (BSMAS) [75], which assesses six behavioral addiction dimensions (e.g., salience and mood modification). Items were rated on a 5-point scale (1 = very rarely; 5 = very often); a sample item is “I spend increasing amounts of time on social media”. The Chinese version was validated using Chinese samples [69]. In this study, McDonald’s ω was 0.91.

### 3.3. Procedure

The protocol was approved by the Ethics Committee of Tongji Medical College, Huazhong University of Science and Technology (Approval No.: [2025] Ethics Review S049) on 23 April 2025. The study was conducted in strict accordance with the ethical guidelines outlined in the Declaration of Helsinki, ensuring the protection of participants’ rights and the integrity of the research process.

Data were collected via an online questionnaire administered in May 2025 using Credamo, a professional survey platform with over three million verified users. A stratified random sampling method was employed to recruit currently enrolled university students over the age of 18 from 30 provinces across mainland China, based on region (Eastern, Central, and Western), gender, and educational level (Junior College to Doctorate), to ensure diversity and representativeness.

Prior to participation, all respondents provided electronic informed consent after being informed of the study’s purpose, data confidentiality, and voluntary nature. Following informed consent, participants were directed to an online questionnaire administered by Credamo, wherein they were asked to report on demographic information, insecure attachment, fear of negative evaluation, loneliness, and social media addiction. The survey took approximately 10 min to complete, and participants received modest compensation for their participation. The questionnaire included attention-check and logic-check items to ensure data quality. Responses were excluded if participants failed two or more attention checks or provided contradictory answers (e.g., indicating both “never use social media” and “more than six hours per day”). Of the 525 responses collected, 400 valid cases remained after excluding 125 invalid entries.

### 3.4. Data Analysis

A priori power analysis using G*Power 3.1 (*α* = 0.05, *power* = 0.80, and *f^2^* = 0.05) indicated a minimum required sample size of 295 for the planned serial mediation analysis (PROCESS Model 6) with seven predictors. The final sample of 400 met this requirement, ensuring adequate statistical power.

Internal consistency reliability was assessed using McDonald’s omega (*ω*) and composite reliability (*CR*) [76,77]. All indices exceeded the acceptable threshold (*ω* = 0.91–0.95; *CR* = 0.87–0.94), indicating satisfactory reliability. Convergent validity was examined using the average variance extracted (*AVE*) and standardized factor loadings, with *AVE* values ranging from 0.54 to 0.65 and standardized factor loadings between 0.70 and 0.88, thus meeting recommended thresholds [76].

To address common method bias, Harman’s single-factor test showed the first factor accounted for 36.58% of the variance, below the 40% threshold [78]. All values of the variance inflation factor (VIF) were below 5, indicating no multicollinearity concerns [79].

Analyses were conducted using SPSS 26.0. Procedures included descriptive statistics, Pearson correlations, and serial mediation analyses based on the PROCESS macro (Model 6), which specifies a chained mediation model with two mediators arranged sequentially and tested using 5000 bootstrap samples. Mediation effects were considered significant when 95% confidence intervals excluded zero [80]. Gender, academic pressure, and average daily social media use were controlled in all models.

## 4. Results

### 4.1. Descriptive Analysis and Correlations

To examine the distributions and interrelationships among the main variables, descriptive statistics and Pearson correlation analyses were conducted for attachment anxiety (AAX), attachment avoidance (AAV), fear of negative evaluation (FNE), loneliness (LNL), and social media addiction (SMA). As shown in Table 2, the mean scores and standard deviations were as follows: AAX (*M* = 3.61, *SD* = 0.93), AAV (*M* = 2.38, *SD* = 0.92), FNE (*M* = 3.42, *SD* = 0.92), LNL (*M* = 2.40, *SD* = 0.76), and SMA (*M* = 3.09, *SD* = 0.80).

Correlation analyses revealed several significant associations. AAX was positively correlated with FNE (*r* = 0.74, *p* < 0.01), LNL (*r* = 0.51, *p* < 0.01), and SMA (*r* = 0.55, *p* < 0.01), and negatively correlated with AAV (*r* = −0.26, *p* < 0.01). AAV showed a weak positive correlation with LNL (*r* = 0.11, *p* < 0.05), but was not significantly correlated with SMA. FNE was positively associated with both LNL (*r* = 0.55, *p* < 0.01) and SMA (*r* = 0.63, *p* < 0.01). LNL was also significantly related to SMA (*r* = 0.57, *p* < 0.01).

Taken together, these results suggest robust associations between insecure attachment dimensions, emotional vulnerability (as reflected in FNE and loneliness), and social media addiction.

### 4.2. Testing the Serial Mediation Effect

To test the proposed serial mediation model, Model 6 of the PROCESS macro for SPSS was employed, using 5000 bootstrap resamples to construct 95% bias-corrected confidence intervals (CIs). After controlling for gender, perceived academic stress, and daily social media usage, the analysis examined the direct, total, and indirect effects of attachment anxiety (AAX) and attachment avoidance (AAV) on social media addiction (SMA) via two mediators: fear of negative evaluation (FNE) and loneliness (LNL) (see Table 3 and Table 4).

AAX showed a significant total effect on SMA, and the direct effect remained significant after including mediators, supporting H1a. AAX positively predicted both FNE and LNL, supporting H2a and H5a, and FNE further predicted LNL, supporting H8. Both FNE and LNL predicted SMA, supporting H3 and H6. All three indirect pathways were significant, supporting H4a, H7a, and H9a. Overall, the indirect effects accounted for 71.4% of the total effect, indicating a clear chained mediation pattern.

AAV showed a significant total effect on SMA in the negative direction, and the direct effect remained negative after including mediators, not supporting H1b. AAV negatively predicted FNE, not supporting H2b, but positively predicted LNL, supporting H5b; FNE further predicted LNL, supporting H8. Both FNE and LNL predicted SMA, supporting H3 and H6. The total indirect effect of AAV on SMA was not significant, indicating no overall indirect association. However, three specific indirect pathways were significant but in opposing directions: a negative path via FNE (supporting H4b), a positive path via LNL (supporting H7b), and a negative chained path via FNE and LNL (supporting H9b). These mixed directions suggest that the indirect effects of AAV offset one another, resulting in a nonsignificant total indirect effect.

Collectively, these results indicate that while AAX exerts a robust and consistent positive influence on SMA through multiple mediators, AAV demonstrates a weaker and more complex pattern, with opposing indirect effects that largely cancel out.

## 5. Discussion

The present study, using data from a sample of Chinese university students, tested a serial mediation model to explore how insecure attachment styles contribute to social media addiction through fear of negative evaluation and loneliness. The findings largely supported the hypotheses and underscore the importance of affective and interpersonal mechanisms in understanding the psychological underpinnings of excessive media use. These results expand the scope of attachment theory within the digital age and offer novel empirical insight into the pathways linking emotional insecurity to maladaptive media behaviors.

### 5.1. Associations Between Insecure Attachment and Social Media Addiction

According to the hypotheses, both attachment anxiety (AAX) and attachment avoidance (AAV) were expected to positively predict social media addiction (SMA), fear of negative evaluation (FNE), and loneliness (LNL). The results partially supported these assumptions. Specifically, AAX was significantly and positively associated with all three variables, in line with theoretical expectations. However, AAV demonstrated a more complex pattern: it positively predicted LNL, but was negatively associated with both FNE and SMA, suggesting a distinct underlying mechanism in the context of media behavior.

Attachment theory posits that individuals with insecure attachment orientations often adopt secondary attachment strategies [81,82]. Anxiously attached individuals typically engage in hyperactivation strategies, characterized by an intense need for closeness and emotional amplification. Due to difficulty in securing stable support in real-life relationships, anxious individuals may turn to social media as a compensatory mechanism for unmet emotional needs. Online interactions offer immediate feedback and a substitute sense of intimacy, which can temporarily alleviate relational anxiety even when real-world connections are unstable [19]. Previous studies also support this view, showing that anxiously attached individuals are more likely to develop addictive tendencies toward platforms that provide instant interpersonal responsiveness [9].

From the perspective of the hyperpersonal model [83], social media environments limit evaluative cues, minimize nonverbal threats, and allow for controlled self-presentation. Such conditions help anxious individuals avoid social threats and maintain a sense of imagined safety. The ability to selectively manage audiences and curate responses enables them to construct an idealized self-image and obtain a form of stable, though virtual, social connection [50]. However, emotional compensation may gradually evolve into dependence and addiction over time [84,85].

In a different vein, avoidantly attached individuals tend to employ deactivation strategies, suppressing emotional expression and avoiding relational vulnerability to maintain autonomy [25]. Their low interest in emotional closeness and general disregard for others’ evaluations may explain the negative associations with FNE and SMA. Delving deeper into this relationship, recent network analysis reveals that the associations between insecure attachment and problematic social media use vary significantly across platforms [86]. Specifically, problematic WeChat use (PWU) is negatively associated with trait attachment avoidance, whereas problematic Sina Weibo use (PSWU) exhibits a positive association with both trait and state attachment avoidance. These differences may be attributed to the varying degrees of privacy and anonymity afforded by each platform. Traditionally, attachment has been conceptualized as a stable personality disposition, often overlooking its capacity for situational variability (i.e., state attachment) [87]. Recent empirical studies suggest that state attachment explains psychological well-being more robustly than trait attachment [88,89]. These findings underscore the necessity for future research to explicitly differentiate between trait and state attachment, and to examine how contextual and platform-specific factors may modulate the activation or suppression of attachment-related processes in digital environments.

### 5.2. Serial Mediational Effects of Fear of Negative Evaluation and Loneliness

This study further examined the mediating roles of fear of negative evaluation (FNE) and loneliness (LNL) in the relationship between insecure attachment and social media addiction (SMA). The findings supported the overall validity of the serial mediation model, but also revealed differences in the structural paths and effect directions between attachment anxiety (AAX) and attachment avoidance (AAV).

In the AAX pathway, both FNE and loneliness served as significant mediators, forming a complete sequential mediation chain. This suggests that anxiously attached individuals may experience an emotional processing sequence of “evaluation sensitivity→loneliness→media dependence” when facing potential social threats. Prior research has shown that individuals high in attachment anxiety are highly sensitive to external evaluation and prone to experiencing FNE [17], which can undermine their confidence in real-world social interactions and increase expectations of rejection—ultimately contributing to heightened feelings of loneliness [56]. In Chinese culture, the heightened emphasis on social evaluation and concern for “face” may further intensify the psychological burden of FNE [67,90]. Consequently, anxious individuals may be more likely to rely on social media platforms that offer lower evaluative risk and greater user control to maintain relationships and present an idealized self. This reliance may provide compensatory emotional gratification and identity affirmation, but also increases the risk of addiction.

Conversely, the AAV pathway exhibited a dual-directional mechanism characterized by offsetting effects. To begin with, AAV negatively predicted FNE, which in turn suppressed SMA, forming two significant inhibitory mediation paths. Although we initially hypothesized a positive association between AAV and FNE, our findings revealed a small negative link. This aligns with the typical deactivation strategies of avoidantly attached individuals, who tend to avoid evaluative pressure and suppress emotional experiences [81], thereby reducing motivations for media engagement. Another possible explanation for the unexpected negative association between AAV and FNE lies in the limitations of self-report measures. Recent psychophysiological studies using pupillometry and wearable sensors indicate that individuals, despite reporting low evaluative anxiety, exhibit heightened autonomic arousal in social evaluative contexts [91,92]. Such findings suggest that defensive deactivation strategies mask rather than eliminate underlying anxiety and highlight the need for multi-method approaches in future research to capture both explicit and implicit aspects of evaluative anxiety. At the same time, AAV also positively predicted LNL and indirectly increased SMA through this compensatory mediation path. This suggests that although avoidant individuals may appear socially detached, their underlying relational needs may still be activated under collectivist cultural expectations of belongingness. When real-world relational resources are lacking, social media may serve as a functional substitute for emotional connection [93].

The coexistence of these negative (inhibitory) and positive (compensatory) pathways constitutes a mechanism offsetting structure, which explains why the total indirect effect of AAV on SMA was not significant. These results also highlight the latent heterogeneity in avoidantly attached individuals’ media use behaviors: while some may reduce media engagement due to social disengagement, others may turn to social media for an alternative connection under cultural or emotional pressures.

### 5.3. Limitations and Future Implications

Despite its theoretical and empirical contributions, this study has several limitations that warrant consideration. First, the cross-sectional design limits causal inference; while the path analysis reveals potential mediation mechanisms, it cannot capture temporal or dynamic processes. Future research should employ longitudinal or experimental designs to clarify causal pathways between attachment orientations, emotional variables, and media use behaviors. Second, the exclusive use of self-report measures may introduce biases such as social desirability and recall error. To enhance ecological validity and reduce common method bias, future studies should incorporate objective indicators, such as behavioral logs, digital usage data, or third-party observations. Although this study employed Harman’s single-factor test and variance inflation factor (VIF) analyses to assess potential common method bias and multicollinearity, it should be noted that these approaches have limited diagnostic power. Future research is encouraged to use more rigorous approaches, such as confirmatory factor analysis, to provide a more comprehensive assessment of method bias. Third, all psychometric scales were originally developed in Western, English-language contexts. Although validated Chinese versions were used, potential issues with translation accuracy, semantic equivalence, and cultural differences in interpretation may remain. Future research should further examine cultural appropriateness and test measurement invariance across cultures. Fourth, the sample was limited to mainland Chinese college students; thus, the findings should be interpreted within the Chinese cultural context. As attachment tendencies and media behaviors are influenced by cultural values, future research should examine diverse cultural groups to assess the cross-cultural relevance of the proposed model or incorporate cultural value measures (e.g., collectivism) to examine potential moderating effects. Moreover, while this study focused on fear of negative evaluation and loneliness as mediators, other psychological variables, such as social comparison, self-esteem, or perceived digital exclusion, may also contribute to social media addiction. Including additional mediators or moderators may help construct a more comprehensive and integrative framework. Finally, nomological validity was not assessed, limiting understanding of how the constructs relate to broader theoretical networks. Future research should evaluate nomological validity to strengthen the model’s construct validity and theoretical integration.

## 6. Conclusions

This study proposed and tested a serial mediation model linking insecure attachment to social media addiction (SMA), with fear of negative evaluation (FNE) and loneliness as mediators. Based on data from Chinese university students, the findings revealed distinct psychological mechanisms for attachment anxiety and avoidance in shaping media use behaviors. Attachment anxiety significantly predicted SMA, primarily through heightened FNE and loneliness, reflecting the role of emotional vulnerability and social approval needs. In contrast, attachment avoidance showed a more complex pattern: while suppressing social concerns reduced SMA risk, underlying loneliness could drive compensatory digital engagement, resulting in offsetting indirect effects.

Theoretically, this study extends attachment theory into the digital domain by identifying emotion-based mediators that explain how attachment orientations influence problematic media use. It contributes to the psychological understanding of SMA by demonstrating that emotional regulation and social cognition jointly mediate this relationship. Moreover, cultural context emerged as a meaningful moderator, suggesting that attachment-related behaviors may manifest differently across socio-cultural settings, which is a valuable insight for integrating cross-cultural and media psychology.

Practically, the results highlight the importance of tailored interventions based on attachment styles. For anxious individuals, interventions such as cognitive–behavioral therapy (CBT) help reduce reliance on digital affirmation [94]. For avoidantly attached individuals, programs that foster emotion recognition and interpersonal skills (e.g., mindfulness-based group interventions) could alleviate emotional isolation and promote healthier offline connections, thereby mitigating excessive media use [95].

Overall, this study provides a solid theoretical foundation and empirical evidence for understanding the emotional and cognitive pathways linking attachment to SMA, offering directions for both future research and intervention design across cultural contexts. Nevertheless, the conclusions should be interpreted in light of limitations related to study design, measurement, and cultural scope, which future research can address through longitudinal, multi-method, and cross-cultural approaches.

## Figures and Tables

**Table 1:** Descriptive statistics (*N*  =  400).

Variable		*n*	(%)
Gender	Male	193	48.25
	Female	207	51.75
Age	18–22	232	58.00
	23–27	168	42.00
Residence	Urban	233	58.25
	Rural	167	41.75
Only child	Yes	127	31.75
	No	273	68.25
Relationship Status	Single	248	62.00
	In a relationship	152	38.00
Education Level	Junior college student	24	6.00
	Undergraduate student	276	69.00
	Master’s	96	24.00
	Doctoral	4	1.00
Academic Pressure (Self-rated)	Very Low	3	0.75
	Low	56	14.00
	Moderate	84	21.00
	High	221	55.25
	Very High	36	9.00
Avg. Daily Social Media Use	<1 h	5	1.25
	[1, 2)	64	16.00
	[2, 4)	176	44.00
	[4, 6)	109	27.25
	≥6 h	46	11.50

**Table 2:** Descriptive statistics and results of correlational analysis of variables (*N*  =  400).

Variable	*M*	*SD*	1.	2.	3.	4.	5.	6.	7.
1. Gender	1.52	0.50	−						
2. Academic Pressure (Self-rated)	3.58	0.87	0.10 *	−					
3. Avg. Daily Social Media Use	3.32	0.92	0.15 **	0.11 *	−				
4. Attachment Anxiety (AAX)	3.61	0.93	0.09	0.33 **	0.17 **	−			
5. Attachment Avoidance (AAV)	2.38	0.92	0.17 **	0.06	−0.02	0.26 **	−		
6. Fear of Negative Evaluation (FNE)	3.42	0.92	0.16 **	0.37 **	0.22 **	0.74 **	−0.07	−	
7. Loneliness (LNL)	2.40	0.76	0.18 **	0.31 **	0.12 *	0.51 **	0.11 *	0.55 **	−
8. Social Media Addiction (SMA)	3.09	0.80	0.23 **	0.38 **	0.45 **	0.55 **	−0.04	0.63 **	0.57 **

Note. Gender was coded as 1 = male and 2 = female; correlations involving gender are point-biserial correlations, while all others are Pearson correlations. * *p* < 0.05; ** *p* < 0.01.

**Table 3:** Direct and total effects of insecure attachment on social media addiction (standardized scores).

Pathway	Estimate	SE	*β*	*t*	Model *R^2^*
Direct Effects					
AAX as the Independent Variables					
AAX→FNE	0.67	0.03	0.68	19.28 ***	0.58
AAX→LNL	0.18	0.05	0.22	3.56 ***	0.34
FNE→LNL	0.28	0.05	0.34	5.41 ***	
AAX→SMA	0.10	0.04	0.12	2.43 *	0.59
FNE→SMA	0.23	0.04	0.26	5.15 ***	
LNL→SMA	0.29	0.04	0.28	6.92 ***	
AAV as the Independent Variables					
AAV→FNE	−0.11	0.05	−0.11	−2.37 *	0.19
AAV→LNL	0.11	0.03	0.13	3.06 **	0.34
FNE→LNL	0.42	0.04	0.51	11.23 ***	
AAV→SMA	−0.06	0.03	−0.07	−2.04 *	0.59
FNE→SMA	0.28	0.04	0.33	7.92 ***	
LNL→SMA	0.32	0.04	0.30	7.68 ***	
Total Effects					
AAX→SMA	0.36	0.03	0.42	10.91 ***	0.49
AAV→SMA	0.07	0.04	−0.08	−1.97 *	0.34

* *p* < 0.05; ** *p* < 0.01; *** *p* < 0.001.

**Table 4:** Indirect and total indirect effects of insecure attachment on social media addiction (standardized scores).

Pathway	Indirect Effect	SE	LLCI	ULCI
Indirect Effect				
AAX→FNE→SMA	0.18	0.04	0.10	0.26
AAX→LNL→SMA	0.06	0.02	0.02	0.11
AAX→FNE→LNL→SMA	0.06	0.02	0.03	0.10
AAV→FNE→SMA	−0.04	0.02	−0.07	−0.01
AAV→LNL→SMA	0.04	0.01	0.01	0.07
AAV→FNE→LNL→SMA	−0.02	0.01	−0.03	−0.00
Total Indirect Effect				
AAX→FNE→LNL→SMA	0.30	0.04	0.22	0.39
AAV→FNE→LNL→SMA	−0.01	0.03	−0.07	0.04

## Data Availability

The raw data supporting the conclusions of this article will be made available by the authors on request due to their sensitive ethical nature.
